# Endolysosomal trafficking regulator SH-BC-893 inhibits coronavirus entry in vitro and in vivo

**DOI:** 10.1038/s41598-026-51335-y

**Published:** 2026-05-11

**Authors:** Brendan T. Finicle, Arielle S. Perrochon, Brandon Chu, Michael J. Buchmeier, Aimee L. Edinger

**Affiliations:** 1 Department of Developmental and Cell Biology, University of California, 2136 Natural Sciences 1, UC Irvine, Irvine, CA 92617 USA; 2 Department of Molecular Biology and Biochemistry, University of California, Irvine, CA USA; 3 Department of Pharmaceutical Sciences, University of California, Irvine, CA USA

**Keywords:** Coronavirus, SARS-CoV-2, Host-targeted anti-viral, Endolysosomal trafficking, PIKfyve inhibitor, MHV-1, Diseases, Drug discovery, Microbiology

## Abstract

SARS-CoV-2 vaccination or infection may not protect from future novel coronavirus outbreaks. Although several drugs targeting essential coronavirus proteins are broadly effective, the error-prone coronavirus RNA-dependent RNA polymerase and a rapid viral replication cycle mean that drug resistance will likely emerge. Small molecules that target sequence-stable host proteins could offer more durable pan-coronavirus activity. Here, we show that the well-tolerated and orally bioavailable small molecule SH-BC-893 protects from endosomal, but not plasma membrane, entry by diverse coronavirus strains. SH-BC-893 alters endolysosomal trafficking similar to PIKfyve inhibitors and, like hydroxychloroquine, reduces cathepsin L activity. While all three compounds block endosomal entry mediated by coronavirus spike proteins in vitro, PIKfyve inhibitors and chloroquine derivatives are ineffective or even increase viral titer in vivo. In contrast, SH-BC-893 reduced viral titer in the lungs of mice infected intranasally with the LD50 of the MHV-1 coronavirus by 3 logs. Thus, poor in vivo outcomes when using apilimod or chloroquine as anti-virals likely reflect pharmacologic limitations of these compounds rather than a flawed therapeutic strategy. We anticipate that SH-BC-893 or optimized analogs with similar tissue pharmacology could control infections by novel coronaviruses, particularly if given in combination with TMPRSS2 inhibitors to block entry at the plasma membrane, an obvious escape pathway. Because it targets host rather than viral proteins, SH-BC-893 might also block infection by many other viruses that enter via endosomal pathways: orthomyxoviruses (influenza), filoviruses (Ebola, Marburg), flaviviruses (Dengue, Zika, and West Nile), alphaviruses (Chikungunya) rhabdoviruses (rabies), and bunyaviruses (Hantaan or Sin Nombre). In sum, further evaluation of SH-BC-893 and/or optimized analogs as potential pan-antiviral agents is merited.

## Introduction

Coronaviruses are enveloped, positive-sense, single-stranded RNA viruses that cause respiratory and gastrointestinal disease in humans and animals. While many coronaviruses produce mild disease, highly pathogenic and/or novel strains for which there is limited pre-existing immunity in human populations have caused significant global health burdens. In 2002, severe acute respiratory syndrome (SARS-CoV) killed an estimated 770 people and infected 8,000 more, a mortality rate of ~ 10%^1^. Ten years later, another deadly coronavirus, MERS-CoV, emerged in the Middle East with an even higher case-fatality ratio of 36%; lower transmissibility between humans and a lower potential for asymptomatic spread kept the MERS-CoV death toll under 1,000^2,3^. In 2019, the novel coronavirus SARS-CoV-2 emerged. SARS-CoV-2 had a lower case-fatality ratio than MERS (~ 1%) but much higher rates of transmission and asymptomatic infection leading to the COVID-19 pandemic and > 7 million deaths worldwide^4^. Novel coronavirus outbreaks are likely to occur in the future as pre-existing immunity from SARS-CoV-2 vaccination or infection is unlikely to protect from newly emergent viruses^5,6^. There is thus a strong rationale for developing broadly effective coronavirus therapeutics.

To date, all FDA-approved preventatives and treatments that limit SARS-CoV-2 replication and infection target viral factors. Vaccines remain the preferred form of prophylaxis due to their safety and effectiveness^7^. Because they induce immunity against viral antigens whose sequences change as new variants emerge, these vaccines need to be updated regularly to maintain peak efficacy, and, like any vaccine, are less effective in immunocompromised individuals^8,9^. Small molecules are available to treat SARS-CoV-2 infections once they occur. Paxlovid combines two drugs: nirmatrelvir inhibits the main SARS-CoV-2 protease and ritonavir slows nirmatrelvir breakdown. In some studies, Paxlovid reduced hospitalizations and deaths when administered within 5 d of symptom onset^10–12^. Similarly, early post-infection treatment with the RNA-dependent RNA polymerase inhibitor remdesivir improves survival among SARS-CoV-2 patients^13,14^. Remdesivir exhibits broad-spectrum antiviral effects against MERS-CoV, Ebola virus, Marburg virus, and other RNA viruses that depend on this virally encoded enzyme^15–17^. While Paxlovid and remdesivir have saved lives, resistant strains are likely to emerge due to the relatively high mutation rate in viruses with RNA genomes^18–21^. Indeed, the long-term value of any coronavirus treatment that targets a viral protein is likely to be limited by the emergence of resistant strains.

**Fig. 1:**
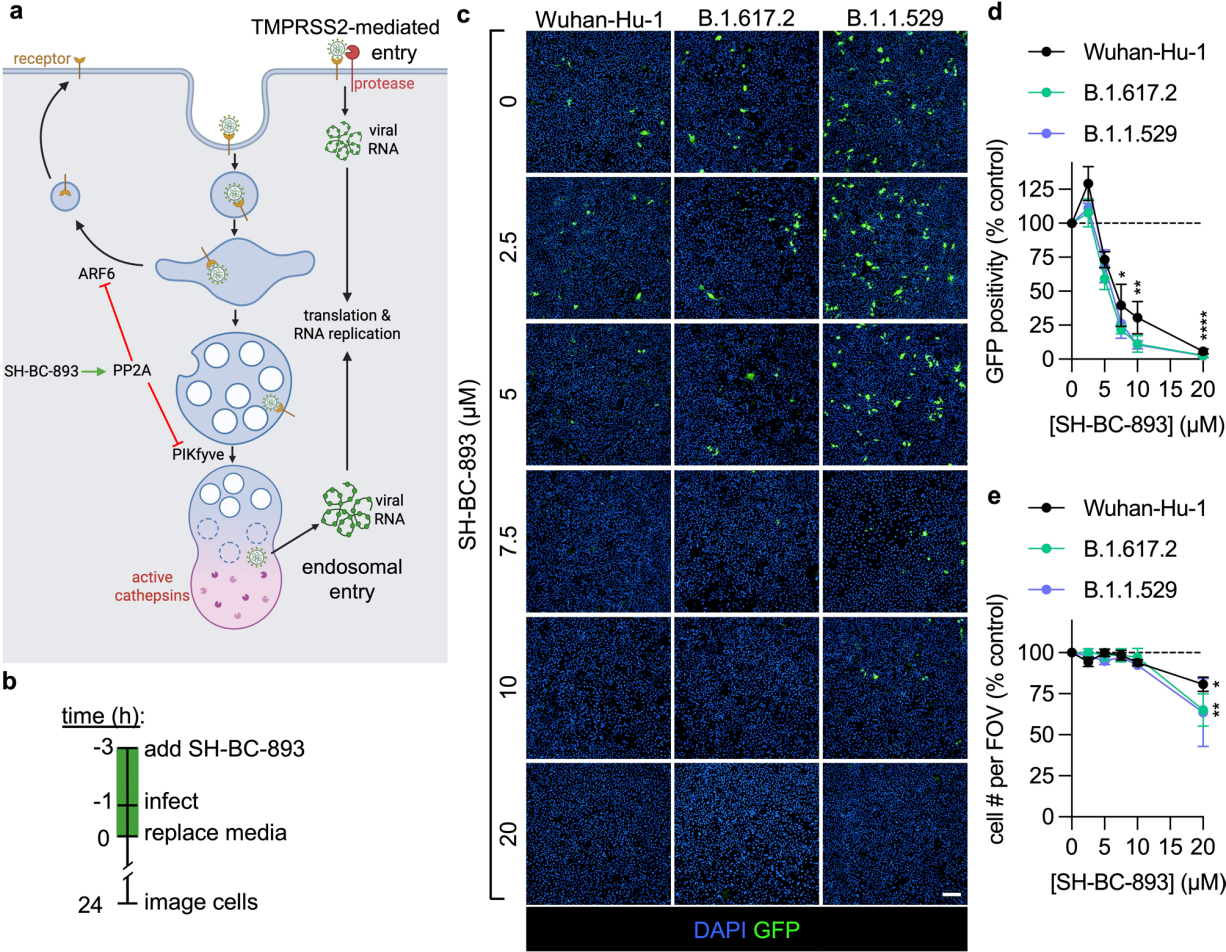
SH-BC-893 prevents infection with multiple SARS-CoV-2 pseudovirus variants. (**a**) Model depicting endosomal entry and TMPRSS2-dependent cell surface entry of coronaviruses. (**b**) Timeline for treatment in (**c**,**d**). (**c**) VeroE6 cells were treated with GFP-reporter pseudoviruses bearing SARS-CoV-2 spike proteins from Wuhan-Hu-1, B.1.617.2, or B.1.1.529 strains and imaged 24 h after infection. Scale bar = 250 μm. (**d**) GFP positive cells at each [SH-BC-893] in (**c**) and normalized to each untreated control. Mean ± SD shown, *n* = 3. Within each strain, each [SH-BC-893] was compared to untreated control using an ordinary one-way ANOVA with Dunnett’s test for multiple comparisons, ****, *p* < 0.0001; **, *p* < 0.01; *, *p* < 0.05. For clarity, comparisons shown on graph represent the lowest significance achieved among the three strains. Unmarked comparisons are *p* > 0.05. (**e**) Number of cells (as defined by individual DAPI-positive nuclei) from images in (**c**) graphed relative to untreated control. Mean ± SD shown, *n* = 3. One-way ANOVA was performed as in (**d**), at 20 µM SH-BC-893 *p* < 0.05 (*) for Wuhan-Hu-1 and *p* < 0.01 (**) for B.1.617.2 and B.1.1.529. Unmarked comparisons are *p* > 0.05. For (**c**–**e**), 5–10 fields of view from 3 independent experiments were quantified and a representative image shown.

Treatments that target sequence-stable host cell proteins may offer broad and more durable protection against coronaviruses. Drugs targeting cellular proteins required for viral entry, assembly, or release could be effective, but blocking viral entry may provide the greatest benefits by stopping viruses before they damage host cells. Inhibitors of TMPRSS2, a host cell protease that facilitates SARS-CoV-2 entry at the plasma membrane, did not provide clear benefits in human trials^22^. This strategy suffers from the limitation that, if spike protein cleavage at the plasma membrane is blocked, coronaviruses readily enter cells by the endosomal route (Fig. 1a). Inhibitors that target endosomal entry have been effective in vitro but likewise failed to control SARS-CoV-2 infection in vivo (e.g. hydroxychloroquine^23–26^ or are too toxic to develop for human use (e.g. bafilomycin A1^27,28^). PIKfyve inhibitors that limit endosome-lysosome fusion were well-tolerated in Phase I clinical trials and showed great promise in vitro but worsened infections in mice and failed to show a benefit in SARS-CoV-2 infected humans^29–34^. In summary, many studies have suggested that while effective in vitro, targeting endosome/lysosome trafficking does not reduce viral entry and spread in vivo. We propose that these failures may reflect pharmacologic limitations of the drugs rather than a flawed strategy. Here, we show that the well-tolerated, orally bioavailable small molecule SH-BC-893 that has been shown to inhibit endosome-lysosome fusion in vivo^35–37^ blocks endosomal entry in vitro, but also dramatically reduces viral titer in the lungs of mice infected with a murine coronavirus strain that produces a severe acute respiratory syndrome^38^.

## Results

### SH-BC-893 blocks endosomal entry mediated by the spike proteins of multiple SARS-CoV-2 strains

SH-BC-893^39^ reduces both endosome-lysosome fusion and ARF6-dependent endocytic recycling (Fig. 1a)^35,36,40^, two trafficking steps reported to be required for SARS-CoV-2 infection^30,41–43^. To determine the extent to which SH-BC-893 inhibits SARS-CoV-2 entry, lentiviruses pseudotyped with spike proteins from the original Wuhan-Hu-1 variant that started the pandemic, the Delta variant (B.1.617.2) that was dominant in 2021, and the Omicron variant (B.1.1.529) that emerged in late 2021 were generated^44^; currently circulating strains are predominantly derived from the Omicron lineage. These assays measure coronavirus spike protein-mediated entry independent of downstream replication events. VeroE6 cells were used as targets for infection because they naturally express ACE2 at levels that support SARS-CoV-2 entry^45^. VeroE6 cells were treated with increasing doses of SH-BC-893 for 3 h prior to infection with lentiviruses bearing a GFP reporter gene and pseudotyped with SARS-CoV-2 spike proteins (Fig. 1b). Cells were washed after 1 h to remove both residual virus and SH-BC-893, and GFP-positive infected cells detected 24 h later (Fig. 1c). At non-toxic concentrations that we have shown inhibit both lysosomal fusion and ARF6-dependent endocytic recycling^35,36,40^, SH-BC-893 blocked infection mediated by the spike proteins of all three SARS-CoV-2 variants (Fig. 1c–e) in VeroE6 cells where these pseudoviruses enter via the endosomal route.

**Fig. 2:**
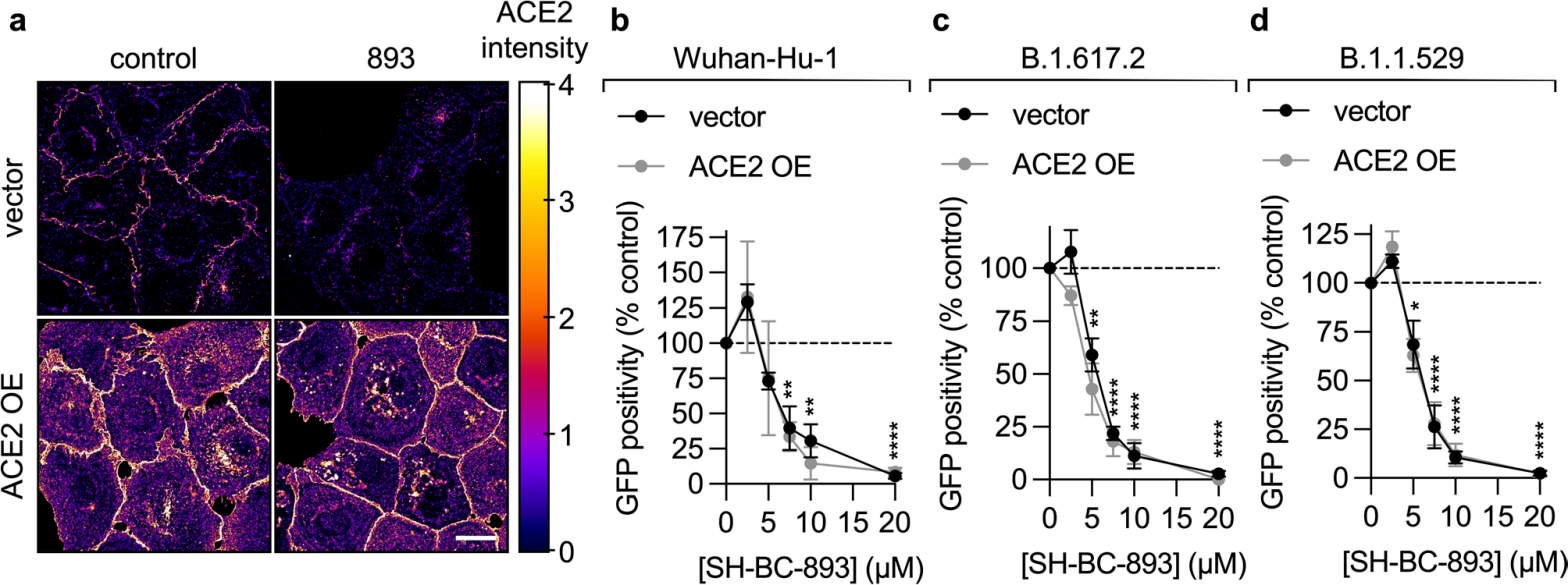
SH-BC-893 does not inhibit SARS-CoV-2 pseudotype infection by reducing cell surface levels of ACE2. (**a**) VeroE6 cells with or without exogenous ACE2 expression (OE, over-expression) were treated with SH-BC-893 (10 µM) for 3 h and stained with antibodies to ACE2. Scale bar = 20 μm. (b-d) VeroE6 cells from (**a**) treated with indicated concentrations of SH-BC-893 for 3 h prior to 1 h infection with GFP-reporter pseudoviruses bearing SARS-CoV-2 spike proteins from the Wuhan-Hu-1 (**b**), B.1.617.2 (**c**), or B.1.1.529 (**d**) strains. Cells imaged 24 h after infection and the percent of cells that were GFP positive quantified and normalized to untreated control at each [SH-BC-893]. Mean ± SD shown, *n* = 3. For (**b**–**d**), 5–10 fields of view from 3 independent experiments are quantified and representative images shown. For both vector and ACE2 OE cells, each [SH-BC-893] was compared to untreated control using an ordinary one-way ANOVA with Dunnett’s test for multiple comparisons with the lowest significance shown at each [SH-BC-893], ****, *p* < 0.0001; **, *p* < 0.01; *, *p* < 0.05. Comparisons shown on graph b represent the significance for vector relative to control. For unmarked comparisons, *p* > 0.05.

The SARS-CoV-2 spike protein uses ACE2 as a primary receptor; binding to ACE2 enables proteolytic cleavage of spike protein triggering a conformational change that mediates viral entry^46^. SH-BC-893 down-regulates cell surface nutrient transporters by interfering with ARF6-dependent recycling^35,40,47,48^. It was possible that blocking ARF6-dependent recycling also reduces surface levels of ACE2, which would limit viral entry by preventing receptor binding. ARF6 knockdown blocks SARS-CoV-2 infection without reducing ACE2 mRNA or total protein levels, findings that would be consistent with reduced surface expression due to inhibition of ACE2 recycling^41^. SH-BC-893 reduced surface levels of endogenous ACE2 in VeroE6 cells with kinetics that could explain the reduced pseudovirus infection (Figs. 1b–d and 2a). To determine whether ACE2 down-regulation from the cell surface was necessary for SH-BC-893 to limit entry, infections were repeated in ACE2 over-expressing cells that maintained high ACE2 surface levels even in the presence of SH-BC-893 (Fig. 2a). SH-BC-893 reduced entry mediated by all three SARS-CoV-2 spike proteins with equal potency in the ACE2 over-expressing and control VeroE6 cells (Fig. 2b–d). These results indicate that SH-BC-893 does not reduce viral entry by limiting access to the ACE2 receptor, at least under these experimental conditions.

**Fig. 3:**
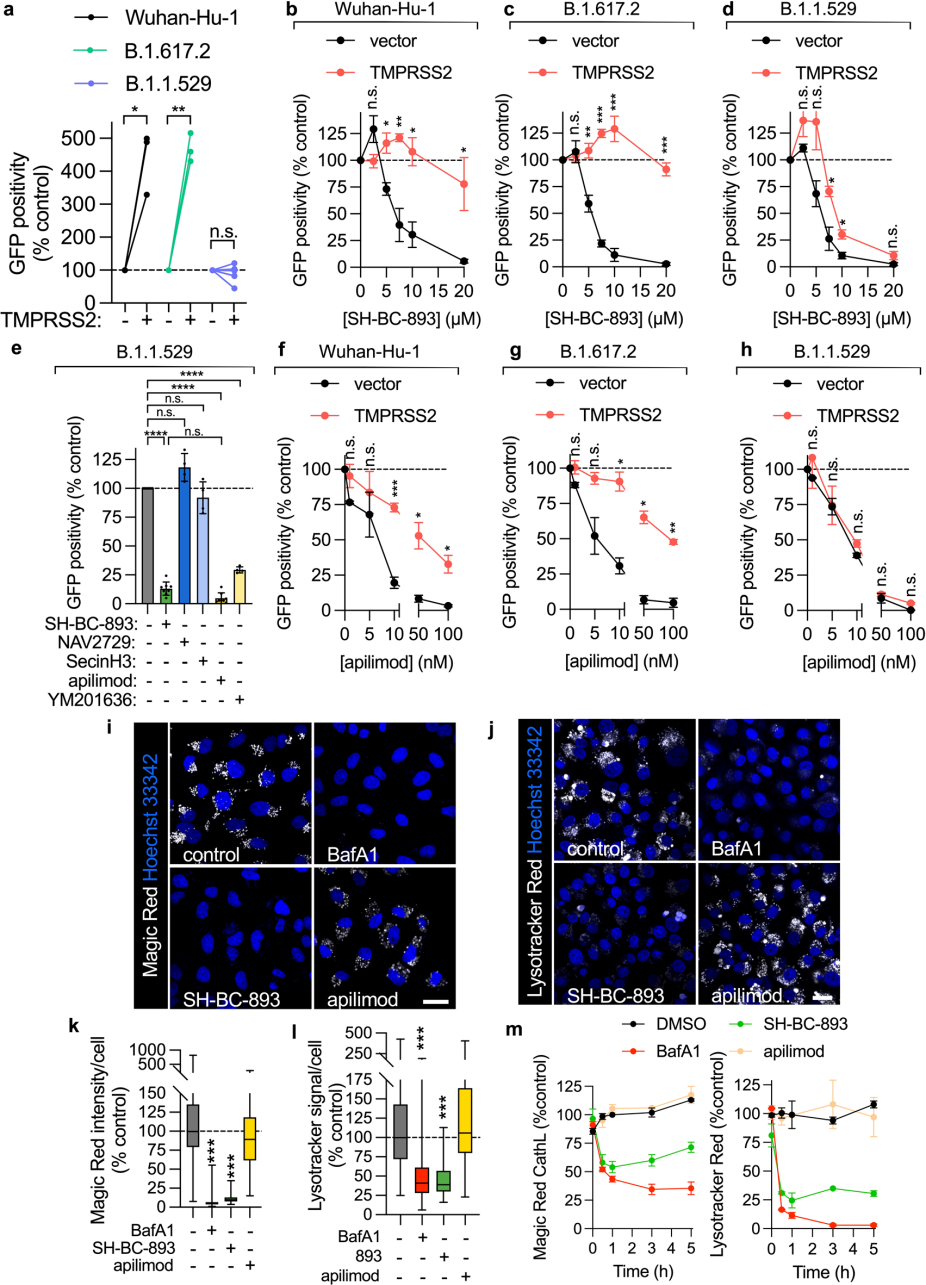
SH-BC-893 selectively blocks endosomal entry by SARS-CoV-2 pseudotypes. (**a**) VeroE6 cells without or with ectopic expression of TMPRSS2 were infected for 1 h with GFP-reporter lentiviruses pseudotyped with spike proteins from Wuhan-Hu-1, B.1.617.2, or B.1.1.529 SARS-CoV-2 strains and imaged 24 h after infection. Percent of cells that are GFP positive was quantified and normalized to each untreated control. Mean ± SD shown, *n* = 3–5. GFP positivity was compared in vector or TMPRSS2 over-expressing VeroE6 cells using a Welch’s t-test to account for unequal SD, **, *p* < 0.01; *, *p* < 0.05; n.s. *p* > 0.05. (**b**–**d**) Same as (**a**), except cells treated with indicated concentrations of SH-BC-893 3 h prior to infection with the indicated pseudovirus. For (**b**–**d**), GFP positivity at each [SH-BC-893] in vector vs. TMPRSS2-expressing VeroE6 cells was compared using a Welch’s t-test to account for unequal SD, ***, *p* < 0.001; **, *p* < 0.01; *, *p* < 0.05; n.s., *p* > 0.05. (**e**) Percent of VeroE6 cells that were GFP-positive 24 h after infection when treated with SH-BC-893 (10 µM), NAV2729 (12.5 µM), SecinH3 (30 µM), apilimod (50 nM), or YM201636 (800 nM) for 3 h prior to 1 h infection with pseudoviruses bearing SARS-CoV-2 spike protein from the B.1.1.529 (Omicron) strain. Mean ± SD shown, *n* = 4–8. A 1-way Brown-Forsythe and Welch ANOVA with Dunnett’s correction for multiple comparisons for (**e**); ****, *p* < 0.0001; n.s., *p* > 0.05. (**f**–**h**) Same as (**b**–**d**), except cells treated with indicated concentrations of apilimod for 3 h before a 1 h infection with pseudovirus. Percent of cells that are GFP positive quantified and normalized to each untreated control. Mean ± SD shown, *n* = 3. GFP positivity at each [apilimod] was compared in vector vs. TMPRSS2-expressing VeroE6 cells using a Welch’s t-test to account for unequal SD. ***, *p* < 0.001; *, *p* < 0.05; n.s., *p* > 0.05. For (**b**–**d**,**f**–**h**), 5–10 fields of view from at least 3 independent experiments are quantified. (**i**) VeroE6 cells were pre-treated with bafilomycin A1 (50 nM), SH-BC-893 (7.5 µM), or apilimod (50 nM) for 3 h then stained with Magic Red cathepsin L substrate for 1 h and imaged. Scale bar = 30 μm. (**j**) VeroE6 cells treated as in (**i**) but stained with Lysotracker Red. (**k**) Quantification of Magic Red signal in (**i**), mean fluorescence signal from 44–81 cells is reported, normalized to the DMSO-treated control. Kruskal-Wallis test with correction for multiple comparisons, ***, *p* < 0.001. (**l**) Quantification of Lysotracker Red signal in (**j**), signal from 127–191 cells is analyzed as in (**k**). Scale bar = 30 μm. (**m**) DBT cells pre-treated with vehicle, bafilomycin A1 (50 nM), SH-BC-893 (5 µM), or apilimod (50 nM) for 0.5, 1, 3, or 5 h prior to staining with Magic Red or Lysotracker Red in the last 30 min of incubation. Fluorescence intensity was measured via flow cytometry, background-subtracted mean fluorescence intensity (MFI) is expressed as percent control. Mean of 2 independent experiments is shown, error bars denote range.

Whether SH-BC-893 also blocks entry at the plasma membrane was next examined. After ACE2 binding, SARS-CoV-2 spike protein becomes susceptible to proteolytic cleavage by either TMPRSS2 at the cell surface or cathepsin L in acidified endosomes (Fig. 1a and^46^. VeroE6 cells do not express TMPRSS2, and thus SARS-CoV-2 entry occurs solely through the endosomal route. SH-BC-893 blocked endosomal entry (Figs. 1b–e and 2b–d). To determine whether SH-BC-893 was equally effective at blocking viral entry at the plasma membrane, VeroE6 cells over-expressing human TMPRSS2 were evaluated. Consistent with published studies^49,50^, TMPRSS2 expression in VeroE6 cells increased the entry of viruses pseudotyped with the Wuhan-Hu-1 and Delta (B.1.617.2) SARS-CoV-2 spike proteins by 4–5-fold (Fig. 3a). Omicron variants contain mutations near the TMPRSS2 cleavage site that reduce processing^44,51–54^. As expected, TMPRSS2 expression did not increase entry of virus pseudotyped with the Omicron spike protein over what was seen in control VeroE6 cells (Fig. 3a). Although SH-BC-893 was effective against Wuhan-Hu-1 and B.1.617.2 pseudoviruses in the absence of TMPRSS2 (Figs. 1c and d and 2b and c), it no longer inhibited entry mediated by spike proteins derived from these strains when TMPRSS2 was present (Fig. 3b, c). In contrast, SH-BC-893 inhibited infection of VeroE6 cells by pseudoviruses bearing the Omicron spike B.1.1.529 to a similar extent in the presence or absence of TMPRSS2 (Figs. 1d and 2d, and 3d). Although the related molecule sphingosine has been suggested to disrupt interactions between spike protein and ACE2^49,55^, SH-BC-893 does not block ACE2-dependent entry at the plasma membrane (Fig. 3b, c). These results (Fig. 3b–d) confirm that the inhibitory effect of SH-BC-893 on viral entry is restricted to the endosomal entry route, at least in VeroE6 cells.

SH-BC-893 simultaneously inhibits endolysosomal trafficking and ARF6-dependent endocytic recycling (Fig. 1a)^35,36,40^. To determine whether one of these cellular effects was sufficient to account for the ability of SH-BC-893 to inhibit viral entry in VeroE6 cells, the effect of SH-BC-893 on Omicron spike-mediated entry was compared to that of PIKfyve inhibitors (apilimod and YM201636) that produce a similar late endosomal trafficking defect and to ARF6 inhibitors (NAV2729 and SecinH3) that restrict endocytic recycling. At concentrations we have shown are sufficient to reduce ARF6-GTP levels and endocytic recycling^40,56^, neither ARF6 inhibitor reduced infection by Omicron pseudotypes (Fig. 3e). In contrast, both PIKfyve inhibitors reduced infection to a similar extent as SH-BC-893. Moreover, apilimod and SH-BC-893 showed the same pattern of entry inhibition among these three SARS-CoV-2 strains, inhibiting entry from endosomes but not TMPRSS2-mediated entry at the cell surface (Fig. 3b–h)^43,57^. Notably, although it produces a similar vacuolation phenotype to PIKfyve inhibitors, SH-BC-893 does not directly inhibit PIKfyve kinase activity or reduce PI(3,5)P_2_ levels but rather alters lysosomal trafficking by activating protein phosphatase 2 A^35,36,40,58,59^. SARS-CoV-2 entry via the endosomal route requires processing of the spike protein by lysosomal proteases such as cathepsin L^60,61^. By altering endosomal trafficking, it was possible that SH-BC-893 and/or PIKfyve inhibitors reduced cathepsin L activity either by altering cathepsin L trafficking or by inhibiting late endosome acidification. Consistent with prior reports^62,63^, apilimod did not reduce cathepsin L activity as measured by cleavage of Magic Red Cathepsin L substrate or endolysosomal acidification in VeroE6 cells as measured by Lysotracker Red (Fig. 3i–l). In contrast, SH-BC-893 reduced cathepsin L activity and lysosomal acidification to a similar extent as the V-ATPase inhibitor bafilomycin A1. Repeating these assays in DBT murine astrocytoma cells that support infection by murine hepatitis virus (MHV) and are commonly used to propagate and titer MHV strains yielded similar results (Fig. 3m). These results suggest that SH-BC-893 can block viral entry both by preventing viral particles from reaching the cathepsin L containing endocytic compartment similar to PIKfyve inhibitors^35,36^ and by reducing cathepsin L activity.

### SH-BC-893 blocks a spreading infection of replication-competent MHV in vitro

**Fig. 4:**
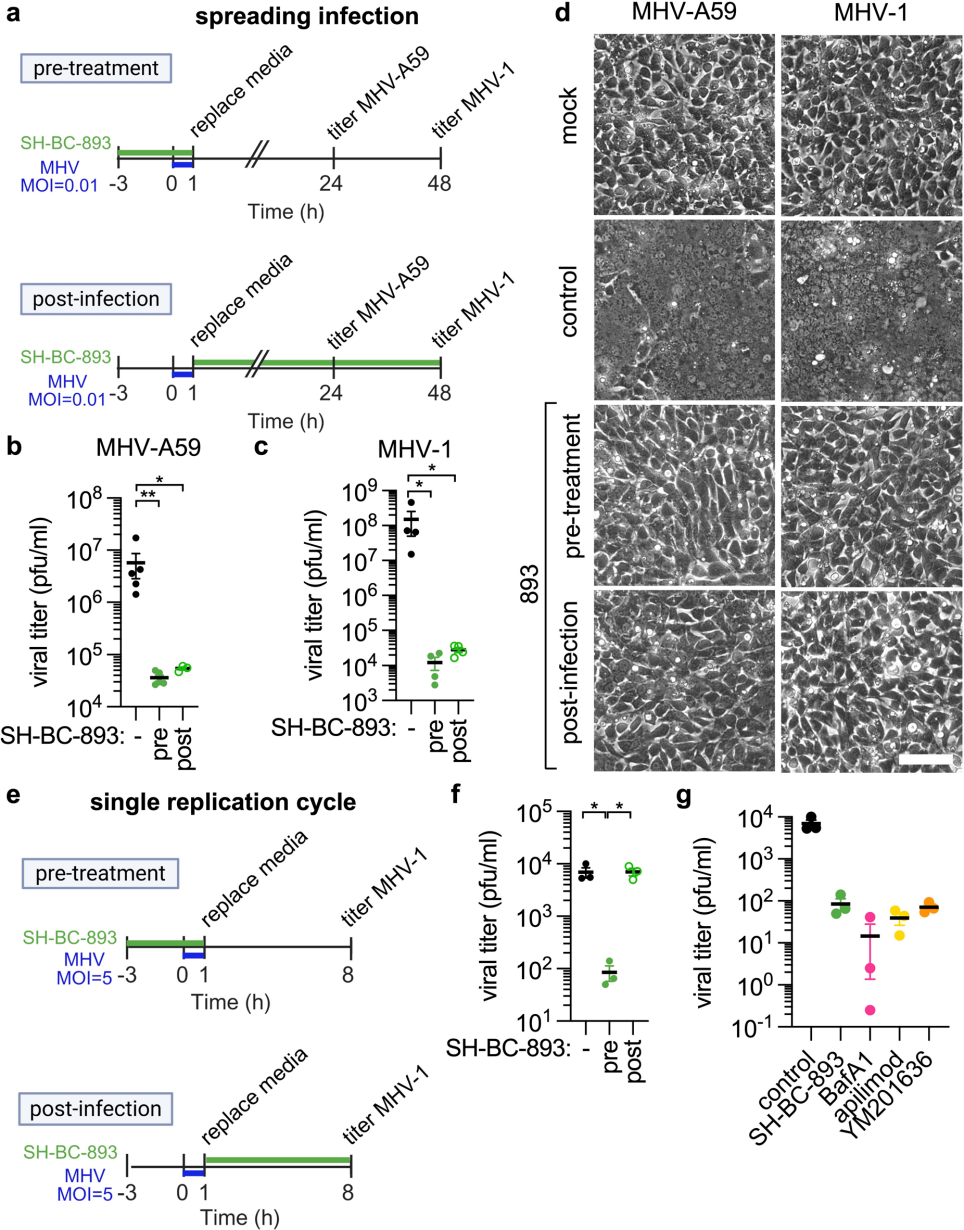
SH-BC-893 inhibits spreading infections of murine hepatitis virus (MHV) in vitro. (**a**) Timeline for prophylactic or post-infection treatments in (**b**). (**b**) Viral titer calculated from a plaque formation assay in DBT cells treated with SH-BC-893 (5 µM) for 3 h prior to a 1 h infection with MHV-A59 at a MOI of 0.01, or cells treated with SH-BC-893 (5 µM) after 1 h of infection. MHV-A59 supernatant collected for titering at 24 h post infection (hpi). Statistical differences across the three conditions was assessed via Kruskal-Wallis H test with post-hoc Mann-Whitney U comparisons, **, *p* < 0.01; *, *p* < 0.05. (**c**) As in (**b**), except using MHV-1 at an MOI of 0.01. MHV-1 supernatant was collected for titering at 48 hpi. Statistical differences across the three conditions was assessed as in (b); *, *p* < 0.05. (**d**) Phase contrast images of DBT cells from the experiments in (**b**,**c**). Scale bar = 100 μm. (**e**) Timeline for prophylactic or post-infection treatments in (**f**,**g**). (**f**) As in (**b**,**c**), except MHV-1 at a MOI of 5, with supernatant collected for titering at 8 h postinfection. (**g**) Viral titer of MHV-1 after a single round of infection following pre-treatment protocol with SH-BC-893 (10 µM), bafilomycin A1 (50 nM), apilimod (50 nM), or YM201636 (800 nM).

Replication-competent MHV is often used to model the beta coronavirus life cycle. Like SARS-CoV-2, MHV is a positive-strand RNA virus that causes either a respiratory or enteric primary infection with secondary involvement of other organs^64^. MHV infects only mouse cells and thus, unlike SARS-CoV-2, does not require BSL-3 level precautions making this model accessible to most laboratories. The MHV-1 strain causes an acute severe respiratory disease in its natural murine host and may mimic host-pathogen interactions better than other mouse models of coronavirus infection that do not produce respiratory disease as severe as that caused by MERS, SARS-CoV, and SARS-CoV-2 in humans^65,66^. Given that SH-BC-893 targets host rather than viral proteins, MHV infection is an appropriate model to address whether SH-BC-893 can function as a broad-spectrum coronavirus inhibitor. Like SARS-CoV-2, MHV can enter cells at the plasma membrane or through the endosomal pathway depending on viral strain and host cell type^67^. The widely used MHV-A59 strain is liver and weakly CNS tropic, while MHV-1 infects the respiratory tract and causes mild to severe lung disease depending on the strain of mice infected^38,68,69^. Whether SH-BC-893 could limit a spreading infection by MHV-A59 or MHV-1 in vitro was evaluated in DBT murine astrocytoma cells that support infection by both strains. DBT cells were treated with SH-BC-893 either before and during or after a 1 h infection with live virus to mimic a prophylactic or therapeutic application, respectively (Fig. 4a). To determine how SH-BC-893 affects a spreading infection, a low multiplicity of infection (MOI) of 0.01 was utilized to allow multiple rounds of replication before significant levels of cytotoxicity developed. Culture supernatant was collected 24–48 h later and titered by plaque formation assay. SH-BC-893 reduced titers of both MHV-A59 and MHV-1 by ≥ 2-logs using either the prophylactic or post-infection application strategy (Fig. 4b, c). This result is consistent with a block in viral entry given that this assay measures a spreading infection that depends on multiple rounds of viral entry. A block in viral entry would lead to decreased expression of coronavirus proteins including the spike protein, explaining the reduced syncytium formation caused by MHV-A59 or MHV-1 in the presence of SH-BC-893 (Fig. 4d). Thus, SH-BC-893 strongly inhibited a spreading infection of MHV, a beta coronavirus that is closely related to SARS-CoV-2.

Based on results with pseudotyped viruses and reduced cathepsin L activity (Figs. 1, 2 and 3), we expected that MHV infection was blocked at the entry step. To test this hypothesis, the experimental design was altered to evaluate the effect of SH-BC-893 on a single MHV-1 infection cycle by increasing MOI to 5 and titering virus 8 h post-infection, the first time point at which virus release was detected. If SH-BC-893 blocks viral entry but not replication as proposed, a “prophylactic” exposure to SH-BC-893 prior to and during the 1 h infection should reduce viral titers after a single infection cycle, but SH-BC-893 addition after the 1 h infection period should be ineffective if post-entry replication steps are not affected by SH-BC-893 (Fig. 4e). Consistent with our prediction that SH-BC-893 primarily blocks viral entry and has minimal impact on post-entry steps, a prophylactic treatment with SH-BC-893 reduced viral titers by 2-logs (> 99%) after a single round of infection but a post-infection treatment had no effect on the amount of infectious virus recovered (Fig. 4f). Consistent with the primary inhibitory action of SH-BC-893 arising from an entry defect, other agents that restrict endosomal entry, bafilomycin A1 and PIKfyve inhibitors, reduced viral titers to a similar extent as SH-BC-893 after a single round of replication (Fig. 4g). These results indicate that SH-BC-893 can efficiently limit a spreading infection of the MHV coronavirus by blocking viral entry, at least in vitro.

### SH-BC-893 prevents a spreading infection with MHV-1 in vivo

**Fig. 5:**
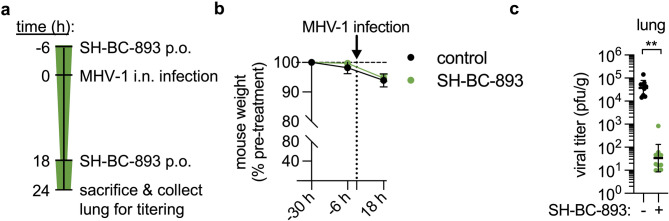
SH-BC-893 reduces MHV-1 viral titers in vivo. (**a**) Timeline of SH-BC-893 treatment and MHV-1 infection of A/J mice. (**b**) Mouse weights 24 h pre-treatment and at the times of SH-BC-893 dosing, expressed as percent control relative to pre-treatment weights. (**c**) Viral titer in the lungs 24 h post-infection. For (**b**,**c**) geometric mean +/- geometric SD, *n* = 9. In (**c**), using a Welch’s t-test to correct for data that is not normally distributed, **, *p* < 0.01.

Although PIKfyve inhibitors and chloroquine robustly block SARS-CoV-2 infection in vitro^29–33^, neither limits coronavirus replication in vivo including in trials in SARS-CoV-2 infected patients^23–26,33,34,68^. These failures might be taken to mean that blocking endolysosomal entry is an ineffective therapeutic strategy. However, the pharmacokinetic properties of these inhibitors are not well defined, and pharmacodynamics in respiratory tissues was not evaluated in the published studies. In contrast, SH-BC-893 pharmacokinetics and pharmacodynamics have been defined^36,37^. SH-BC-893 is effective in mouse models of cancer, obesity, and oligonucleotide delivery with oral dosing at 120 mg/kg with a plasma half-life of 10 h and peak tissue levels reached at 4–8 h post-administration^35–37^. Favorable pharmacodynamics have been established in multiple tissues including the lungs where SH-BC-893 altered endolysosomal trafficking to promote antisense oligonucleotide delivery^36^. SH-BC-893 is well tolerated even with chronic administration. Blood chemistry and complete blood counts were not affected even after 11 weeks of daily administration or after administering twice the effective dose; voluntary wheel running, a holistic measure of mouse health and well-being, was also not reduced by repeated SH-BC-893 administration^35–37^. To determine whether SH-BC-893 could control a respiratory coronavirus infection in vivo, MHV-1 was administered intranasally at the LD50 dose in A/J mice, a strain where MHV-1 induces a SARS-like disease^38,70^. Based on its established tissue pharmacokinetics^36,37^, 120 mg/kg SH-BC-893 p.o. was administered 6 h prior to nasal infection with MHV-1 and again 24 h later (18 h post-infection) (Fig. 5a). Mice were weighed prior to each treatment (Fig. 5b) and sacrificed 24 h after infection when viral titers peak^68^. Consistent with in vitro results with MHV-1 (Fig. 4), SH-BC-893 reduced viral titer in the lungs by more than 3 logs (Fig. 5c). These results suggest that blocking endolysosomal entry in host cells can be an effective strategy to control a respiratory coronavirus infection in vivo.

## Discussion

Here we show that the small molecule SH-BC-893 blocks the endosomal entry of multiple SARS-CoV-2 pseudotyped lentiviruses and MHV strains. SH-BC-893 has multiple actions that might contribute to a block in coronavirus entry: reducing ARF6-dependent endosome recycling, inhibiting endosome-lysosome fusion, and reducing cathepsin L activity (Fig. 3i–m and^30,35,40–43,58^. Although SH-BC-893 may reduce plasma membrane ACE2 levels via ARF6 inhibition (Fig. 2a^40–42^), this was not the mechanism by which SH-BC-893 limited infection in VeroE6 cells (Fig. 2b–d). ACE2 down-regulation may be a more important mode of inhibition in cells that express low levels of ACE2 or MHV receptors. Consistent with the conclusion that disrupting ACE2 recycling was not the primary mechanism of anti-viral action for SH-BC-893, two structurally distinct ARF6 inhibitors, NAV2729 and SecinH3, failed to inhibit entry by pseudoviruses bearing the Omicron spike protein at concentrations that reduce ARF6-GTP levels (Fig. 3e and^40,56^. In contrast, PIKfyve inhibitors apilimod and YM201636 inhibit lysosomal trafficking like SH-BC-893 and reduced viral titers to a similar extent as SH-BC-893 (Figs. 3e–h and 4g and^35,36^. Unlike PIKfyve inhibitors, SH-BC-893 reduces cathepsin L activity and increases endolysosomal pH to a similar extent as bafilomycin A1 (Fig. 3i–m), parallel activities that would also limit entry from late endocytic compartments (Fig. 4g). These potentially redundant effects should make SH-BC-893 an effective entry inhibitor. Consistent with results from entry assays using SARS-CoV-2 spike pseudotyped viruses, SH-BC-893 blocked MHV infection at the entry step (Fig. 4e, f). This block could be explained by its ability to limit endolysosome fusion, reduce cathepsin L activity, or both (Fig. 4g). It remains unclear how SH-BC-893 elevates lysosomal pH, but no direct interactions with V-ATPase subunits were detected in rigorous chemoproteomics assays or using a photoaffinity labeling probe in intact cells^58^. Because SH-BC-893 did not reduce MHV titer when added after virus during a single replication cycle (Fig. 4e, f), post-entry replication steps were not inhibited under these experimental conditions. SH-BC-893 has multifaceted effects on endolysosomal trafficking and nuclear import^58^, and it remains possible that SH-BC-893 could lead to cellular responses that compromise viral replication or release if viruses manage to circumvent the entry block. It is also possible that the replication and release of viruses other than MHV-1 may be more sensitive to the actions of SH-BC-893. Although other agents that modulate endolysosomal trafficking and/or cathepsin activity have already been shown to inhibit coronavirus entry as effectively as SH-BC-893 in vitro (Fig. 4g and^29–32^, our study breaks new ground by establishing that SH-BC-893 also dramatically reduces viral titers in vivo.

PIKfyve inhibitors and hydroxychloroquine that block endosomal entry by both coronaviruses and Ebola virus in vitro are ineffective or even worsen infections when used in vivo^29–34,71^. For example, administering the maximum tolerated dose of the PIKfyve inhibitor WX8 1 d prior to intranasal infection with SARS-CoV-2 B.1.351 increased rather than decreased the viral titer in lung homogenates 10-fold at 2 d post-infection and 100-fold at 4 d post-infection^33^. Prophylactic dosing with the related PIKfyve inhibitor NDF also increased viral titer in the lungs of infected mice at 4 d post-infection. When tested as a post-infection treatment in mice challenged with the mouse adapted SARS-CoV-2 MA-10^66^, PIKfyve inhibitors were ineffective (WX8), increased the viral load (NDF and apilimod), or reduced survival (apilimod)^33^. Apilimod was similarly ineffective in a human clinical trial (NCT04446377^34^) in adults that tested positive for SARS-CoV-2 infection; no difference in viral load or hospitalization/death was reported between treatment and placebo groups. Many studies show that hydroxychloroquine can block endosomal infection in vitro, but it is also ineffective or even contraindicated as an anti-viral agent given worsening disease in some cases^24,71,72^. These negative results with host-targeted drugs that block endolysosomal entry suggested that this strategy could not control viral infections under in vivo conditions. Our results with SH-BC-893 suggest that targeting endolysosomal trafficking and cathepsin activity could be a viable therapeutic strategy. Importantly, it remains possible that SH-BC-893 has effects on the innate or adaptive immune system or post-entry effects such as activating the integrated stress response in infected cells that might contribute to infection control in vivo even though post-entry inhibition was not observed in time of addition studies in vitro (Fig. 4e, f). Additional experiments will be required to explore these possibilities.

Only some of the reported failures of endolysosomal trafficking inhibitors as anti-virals can be explained by their inability to block entry at the plasma membrane. Like SH-BC-893, PIKfyve inhibitors are only effective against SARS-CoV-2 in vitro in the absence of TMPRSS2 or when strains are not capable of plasma membrane entry^30^. The spike proteins of the B.1.351 (a Beta strain) and mouse-adapted MA-10 strains of SARS-CoV-2 used to evaluate PIKfyve inhibitors in mice were likely susceptible to TMPRSS2 cleavage; plasma membrane entry may have reduced the antiviral effect of PIKfyve inhibitors in vivo. The SARS-CoV-2 strains in circulation in the United States during the in-human apilimod trial conducted between July 2020 and April 2021 were also likely susceptible to TMPRSS2 cleavage; a phenotype that likely rendered them much less sensitive to PIKfyve inhibitors (Fig. 3f–h and^30,34,51,73^. However, the inability to block plasma membrane entry is unlikely to completely account for the failure of PIKfyve inhibitors as antivirals in vivo. Unlike coronaviruses, Ebola virus enters host cells exclusively through the endosomal route as its receptor, NPC1, is only found in late endosomes/lysosomes^74,75^. PIKfyve inhibitors robustly block Ebola virus replication in vitro, but apilimod mesylate was again ineffective in mice infected with a mouse adapted Ebola virus variant^30,63,76,77^. Thus, PIKfyve inhibitors are not effective in vivo even against viruses that are restricted to the endosomal entry pathway. Hydroxychloroquine has a similar profile, blocking endosomal entry in vitro but failing against Ebola virus in vivo^71^. Suboptimal tissue pharmacokinetics might explain or contribute to these in vivo failures. Apilimod mesylate has not been established to hit its target at tolerated doses; it was not efficacious in human Phase II clinical trials for Crohn’s disease^78^ or rheumatoid arthritis^79^ where it failed to alter cytokine levels as expected. Apilimod’s pharmacodynamics were not also not evaluated in mice, but the maximum tolerated doses of PIKfyve inhibitors were ineffective^33^. These caveats introduce uncertainty whether PIKfyve inhibitors and chloroquine are flawed drugs or whether targeting endosomal trafficking was a failed strategy.

In contrast to PIKfyve inhibitors, the pharmacokinetics and pharmacodynamics of SH-BC-893 have been studied^35–37^. As mentioned above, the plasma half-life of SH-BC-893 is 10 h in mice allowing for once daily dosing. Tissue levels peak at 4–8 h post-gavage, with the highest SH-BC-893 levels achieved in the lung^36^. Trafficking-related phenotypes have been observed in multiple tissues (tumor, liver, brain, fat and lung) when mice are dosed with 120 mg/kg p.o^36,37^. Particularly relevant to the current study of viral respiratory tract infection, SH-BC-893 increases antisense oligonucleotide activity in the lungs of treated mice demonstrating that intracellular trafficking is altered by this dose; the tissue with the highest SH-BC-893 levels after repeated dosing was the lung^36^. Importantly, SH-BC-893 is not toxic at 120 mg/kg even when given chronically^35–37^. No signs of organ toxicity were detected (blood chemistry) after 11 weeks of 5 days on/2 days off dosing, and rapidly proliferating cells in the gut (histopathology) and bone marrow (complete blood count) were not affected at doses that produced vacuolation in tumors consistent with inhibition of endosome-lysosome fusion^35^. Rather than being toxic, SH-BC-893 promotes metabolic homeostasis in mice maintained on a high fat diet^37^. In this study, voluntary wheel running, a holistic and sensitive measure of mouse health and well-being^80,81^, was equivalent in vehicle and SH-BC-893-treated (120 mg/kg p.o.) mice dosed every other day for 4 weeks. In contrast to PIKfyve inhibitors, doubling the dose of SH-BC-893 to 240 mg/kg was not toxic, at least in the short term^36^. Together, these published studies establish that SH-BC-893 at 120 mg/kg p.o. blocks endolysosomal trafficking in multiple tissues and is not toxic to even rapidly proliferating tissues, although it will be important to confirm the long-term tolerability of SH-BC-893 in virally infected mice in future studies.

In contrast to results with PIKfyve inhibitors and hydroxychloroquine, the effects of SH-BC-893 were consistent in vitro and in vivo. MHV-1 titers were performed in mice infected with the intranasal LD50 at a time point selected to capture peak viral titers^38^. SH-BC-893 reduced viral titer in the lungs by 3 logs (Fig. 5c), a close match with in vitro results (Fig. 4c). Intranasal MHV-1 infection was selected as the model system because our primary goal was to assess the potential of SH-BC-893 to treat future outbreaks of novel, divergent coronaviruses. MHV-1 infection of A/J mice accurately models the coronavirus-induced severe acute respiratory syndrome induced by MERS, SARS-CoV, and SARS-CoV-2 in humans, including the alveolar disease that is lacking in some mouse models^38^, while avoiding the challenges associated with BSL-3 work (wild type SARS-CoV-2 and mouse adapted strains like MA-10 remain BSL-3 agents despite the availability of an effective vaccine). In the context of a novel coronavirus outbreak, it would be prudent to combine endosomal trafficking regulators like SH-BC-893 with inhibitors of the cell surface proteases that allow plasma membrane entry to disable this obvious resistance pathway (Figs. 1a and 3a-h). TMPRSS2 inhibitors and/or broadly active RNA-dependent RNA polymerase inhibitors might make good choices for combination therapies that would limit the emergence of resistant strains. A combination therapy that includes inhibitors of cellular proteins required for coronavirus assembly^82^ may further suppress the emergence of resistant strains. Although SH-BC-893 was administered to mice prophylactically (before viral infection) in this study to maximize its effect, SH-BC-893 limits a spreading infection even when added after the first round of viral entry (post-infection samples in Fig. 4b–d) suggesting it will also be effective if given post-exposure. In sum, it is likely that SH-BC-893 or optimized analogs could be used to treat infections with novel coronavirus strains that are restricted to endosomal entry or given in combination with a surface protease inhibitor to treat strains that use both plasma membrane and endosomal entry pathways.

Therapeutics that can be used against multiple viruses remain an unmet clinical need. Vaccines for SARS-CoV-2 have saved millions of lives^83^, but pre-existing immunity may not protect against newly emergent strains. The development and mass production of a new vaccine would take time once an outbreak occurs. Small molecules like SH-BC-893 that target host cell factors could be produced in advance of need while also offering added benefits like stability at room temperature and oral bioavailability. It will be important to evaluate the efficacy of SH-BC-893 against a broader range of coronaviruses, its impact on post-infection outcomes, and the optimal dosing regimen in future studies. Therapeutics that are effective against other virus families with high epidemic or pandemic potential would have great value^84^. Many human viruses that enter exclusively through endosomal pathways cause severe or lethal disease: orthomyxoviruses (influenza), filoviruses (Ebola, Marburg), flaviviruses (Dengue, Zika, and West Nile), alphaviruses (Chikungunya), rhabdoviruses (rabies), and bunyaviruses (Hantaan and Sin Nombre viruses). Thus, the potential for SH-BC-893 activity against additional viral families should also be evaluated in future studies.

## Methods

### Cell lines and cell culture

African Green Monkey Kidney Epithelial Cells (Vero E6) Expressing High Endogenous Angiotensin-Converting Enzyme 2 were obtained through BEI Resources, NIAID, NIH (NR-53726). Cells were cultured using DMEM supplemented with 10% FBS and 1 mM sodium pyruvate. Delayed Brain Tumor (DBT) cells were maintained in DMEM supplemented with 10% FBS. All cells were maintained at 37 °C in 5% CO_2_. Cells were tested for *Mycoplasma* every 1–3 months^85^ and maintained in culture for no more than 3 weeks before thawing new, low-passage vials.

### Chemicals and reagents

SH-BC-893 was synthesized by IntelliSyn RD (Montreal, Quebec, Canada). Chemicals were obtained from: apilimod (SelleckChem cat#S6414), NAV2729 (Fisher Scientific, cat#5986), SecinH3 (Cayman Chemicals, cat#10009570), YM201636 (SelleckChem cat#S1219), bafilomycin A1 (Cayman Chemicals, cat#11038). Stock solutions were prepared, aliquoted, and stored at − 20 °C as follows: SH-BC-893 (5 mM in H_2_O), apilimod (100 µM in DMSO), NAV2729 (12.5 mM in DMSO), SecinH3 (30 mM in DMSO), YM201636 (1.6 mM in DMSO), bafilomycin A1 (8 mM in DMSO). Magic Red Cathepsin L assay reagent was purchased from BioRad (cat# ICT-941) or Antibodies Inc. (cat# SKU-941). Lysotracker Red was purchased from Life Technologies (cat# L7528). Hoechst 33342 was purchased from Life Technologies (cat# H3570).

### Plasmids and stable cell line generation

pTRIP-SFFV-Hygro-2 A-TMRPSS2 was a gift from Nir Hacohen (Addgene plasmid #170390; http://n2t.net/addgene:170390; RRID: Addgene 170390). pWPI-IRES-Bla-Ak-ACE2 was a gift from Sonja Best (Addgene plasmid # 154981; http://n2t.net/addgene:154981; RRID: Addgene 154981). Stable cell lines were generated by transducing target cells with lentivirus and drug selection with hygromycin or blasticidin, respectively. pTwist-SARS-CoV-2 Δ18 was a gift from Alejandro Balazs (Addgene plasmid #164436; http://n2t.net/addgene:164436; RRID: Addgene 164436). pcDNA3.3-SARS2-B.1.617.2 was a gift from David Nemazee (Addgene plasmid #172320; http://n2t.net/addgene:172320; RRID: Addgene 172320). pTwist-SARS-CoV-2 Δ18 B.1.1.529 was a gift from Alejandro Balazs (Addgene plasmid # 179907; http://n2t.net/addgene:179907; RRID: Addgene 179907). pLenti CMV GFP Puro (658-5) was a gift from Eric Campeau & Paul Kaufman (Addgene plasmid #17448; http://n2t.net/addgene:17448; RRID: Addgene 17448).

### Pseudovirus assays

Pseudovirus was generated by transfecting 293 T cells with pLenti CMV GFP Puro, psPAX2, and mammalian expression plasmids bearing the spike proteins of Wuhan-Hu-1, B.1.617.2 (Delta), or B.1.1.529 (Omicron). After 48 h, pseudovirus was harvested from 293 T cells and filtered with a 0.45 µM filter (Fisher Sci cat# 09–720-4) to remove floating cells. VeroE6 cells seeded at 8,000 cells per well in a 96 well plate were pre-treated for 3 h with indicated concentrations of SH-BC-893, NAV2729, secinH3, apilimod, or YM201636 before cells were infected with pseudovirus for 1 h. After 1 h, media was aspirated and replaced with virus-free media. GFP positivity was assessed 24 h later. Pseudoviruses were prepared fresh for each experiment.

### MHV methods

Recombinant Murine Coronavirus MHV-A59 with Enhanced Green Fluorescent Protein (eGFP), NR-53,716 and Murine Coronavirus, MHV-1, Plaque Purified, NR-53,715 were obtained through BEI Resources, NIAID, NIH. To generate viral stocks, 2 million DBT cells were plated in a T75 flask. Media was replaced with 5 ml DME2^86^ 20–24 h after seeding and virus added (MOI = 0.001 pfu/cell for MHV-A59, and MOI = 0.1 pfu/cell for MHV-1), incubating on a rocker at room temperature for 1 h. After adding an additional 10 ml of DME2, flasks were returned to the incubator. Cytopathic effect (CPE) was monitored every 12–24 h. Once > 95% of cells were involved in syncytia and ~ 25% of cells had detached (24–36 h for MHV-A59 and 48–72 h for MHV-1) the T75 flask was frozen at −80 °C for at least 1 h. Flasks were then placed in a 37 °C water bath until almost fully thawed, with final thawing occurring at RT. The cell suspension was transferred to a 50 ml conical tube, vortexed 3 × 10 s with a 10 s rest on ice in between. Lysate was clarified by centrifuging at 1,900 x g for 10 min at 4 °C. Supernatant (viral stock) was aliquoted and stored at −80 °C.

To assess the effect of compounds on MHV infection, 300,000 DBT cells/well were seeded in a 6-well plate in DMEM with 10% FBS and used 16–24 h later. Media was removed and cells were washed once with PBS before adding DMEM containing 2% FBS and the virus at the indicated MOI. Plates were rocked gently at RT for 0.5–1 h then the medium was aspirated and washed once with PBS before adding virus-free DMEM containing 2% FBS. Viral supernatant was collected at the indicated timepoints and stored at −80 °C until titering.

Viral stocks were titered in a plaque assay. DBT cells were seeded and infected as described above. A 10-fold serial dilution was generated for each viral supernatant using ice-cold DMEM containing 2% FBS. DBT cells were washed once in PBS before viral dilutions were added. After rocking at RT for 0.5–1 h, the medium was aspirated and replaced with DMEM containing 2% FBS and 1% agarose. Plates were fixed 24–36 h later with 4% formaldehyde for 1 h. After fixation, the agarose overlay was removed and plates stained with 0.01% crystal violet dye dissolved in 70% EtOH for 1 min. Plates were washed 2-3X in ddH_2_O and dried overnight before plaques were manually counted.

### Fluorescence microscopy

VeroE6 cells were plated at 12,000 cells/well in an 8-chamber glass slide (Cellvis, cat# C8-1.5 H-N) and treated 24 h later with 5 µM SH-BC-893 for 6 h, then washed 1X with PBS, fixed with 4% PFA for 10 min, and blocked in PBS containing 10% FBS and 0.3% saponin for 30 min at RT. After blocking, cells were incubated in primary antibody (LEAF Purified anti-human ACE2 Antibody Biolegend cat#503602) overnight at 4 °C and washed 3X with PBS. Cells were incubated with Alexa Fluor594 donkey anti-goat secondary antibody (Invitrogen, cat#A11058) for 30 min at RT then washed 3X with PBS, stained for DAPI for 5 min, washed 1X with PBS, and imaged using ZEN digital imaging software on a Zeiss LSM900 with Airyscan 2 confocal microscope with a Plan-Apochromat 63×/1.40 Oil DIC objective. GFP pseudovirus assays were imaged using a Nikon TE2000 with a CoolSnap camera with a CFI Plan Fluor ELWD DM 20x C objective, n.a. 0.45, w.d. 7 mm. Quantification of microscopy images was performed using ImageJ. The number of GFP positive cells was expressed as a percentage of the total number of cells and normalized to the untreated control. At least 500 cells per experiment from 3 independent experiments were analyzed for each experiment. For cathepsin L activity assays, 15,000 VeroE6 cells were plated in 8-chamber glass bottom slides (CellVis cat# C8-1.5 H-N). Twenty h after seeding, cells were treated for 3 h with compounds, 1 h with Magic Red reagent, washed with PBS, stained with Hoechst 33342, and immediately imaged live on a Zeiss LSM780 confocal microscope with a 63x oil objective. For lysosomal acidifications assays, cells plated in an 8-chamber glass bottom slide were treated with compounds for 3 h. In the last 30 min of treatment, Lysotracker Red (50 nM) and Hoechst 33342 (1 µg/mL) were added. Cells were imaged live on a Zeiss LSM900 confocal microscope with a 63x oil objective. Magic Red or Lysotracker Red signal was measured by creating ROIs around each cell, measuring intracellular mean grey value, subtracting background signal, and normalizing to the median of the control as the data was not normally distributed.

### Flow cytometry

DBT cells were seeded in 24 well plates at 60,000 cells/well. Twenty-four h later, they were treated with compounds for 0.5, 1, 3, or 5 h. In the last 30 min of treatment, Magic Red reagent (1:10 dilution as per manufacturer’s protocol) or Lysotracker Red (50 nM) were added to the medium and cells incubated at 37 °C. Cells were then washed with PBS, trypsinized, and centrifuged at 1,500 rpm for 3 min then resuspended in 200 µl live cell imaging solution (Thermo Fisher cat #A59688DJ). Data was collected using a Becton Dickinson LSR Fortessa X-20 and analyzed with FACSDiva (BD Biosciences).

### Mice

All mouse experiments were approved by the Institutional Animal Care and Use Committee of the University of California, Irvine. All methods were performed in accordance with the relevant guidelines and regulations. Six to eight week old, male A/J mice were purchased from The Jackson Laboratory and acclimated for 7 days prior to experimentation. Mice were housed under a 12:12 h light/dark cycle at 20–22°C in groups of 2. Cages contained 1/8” corncob bedding (7092A, Envigo, Huntingdon, UK) enriched with ∼6 g of cotton fiber nestlets (Ancare, Corp., Bellmore, NY). Mice were fed the vivarium stock diet (chow, 2020x, Envigo). Access to food and water was ad libitum. Mice were weighed and randomized with GraphPad Prism to receive vehicle or SH-BC-893 ensuring that mean body weight was similar in each group. Vehicle or SH-BC-893 was administered orally using polypropylene feeding tubes (20 g × 38 mm; Instech Laboratories Inc., Plymouth, PA) at 120 mg/kg of SH-BC-893 in 5% DMSO, 5% Cremophor, and 16% HPBCD (stock = 24 mg/mL in H_2_O). Feeding tubes were dipped in 1 g/ml sucrose immediately prior to treatment to induce salivation. Mice were anesthetized with 87.5 mg/kg ketamine and 12.5 mg/kg xylazine solution delivered at 5 ml/kg I.P prior to intranasal administration of 500 pfu MHV-1 diluted in serum-free DMEM. SH-BC-893 was given 6 h before and again 18 h after MHV-1. Mice were sacrificed by CO_2_ inhalation followed by cervical dislocation 24 h post-infection. Lungs were snap frozen in liquid nitrogen and stored at −80°C. All animals were included in the analysis. To determine viral titers, lungs were homogenized in DME2 supplemented with 1% P/S using the Next Advance Navy RINO Lysis Homogenizer Pre-Filled Bead Kit (Stellar Scientific cat#NA-NAVYR5) titered as described above.

### Statistical analysis

All experimental data are from ≥ 3 independent biological replicates unless otherwise indicated. Statistical analysis was performed using GraphPad Prism software and Microsoft Excel. Data comparisons between two groups were calculated using t-tests as described in figure legends, comparison between > 2 groups utilized ANOVA as described in figure legends. Corrections for multiple comparisons were made as indicated in the legends and adjusted P-values reported as: n.s., not significant, *p* ≥ 0.05; *, *p* < 0.05; **, *p* < 0.01; ***, *p* < 0.001; ****, *p* < 0.0001; key comparisons are shown in the figures.

## Data Availability

The data generated and analyzed in this study is included in this published article.
